# Effect of parameters on surface roughness during the ultra-precision polishing of titanium alloy

**DOI:** 10.1371/journal.pone.0272387

**Published:** 2022-08-01

**Authors:** Yonggou Lou, Hongbing Wu

**Affiliations:** 1 School of Haitian, Ningbo Polytechnic, Ningbo, China; 2 College of Mechanical and Energy Engineering, NingboTech University, Ningbo, China; 3 Department of Industrial and Systems Engineering, State Key Laboratory in Ultra-precision Machining Technology, The Hong Kong Polytechnic University, Hung Hom, Kowloon, Hong Kong; Semnan University, ISLAMIC REPUBLIC OF IRAN

## Abstract

Titanium alloys have great potential in ultra-precision situations due to the excellent properties, such as high corrosion resistance, high specific-strength and high biocompatibility. However, the application of titanium alloys in ultra-precision field is limited by the poor machinability. There are difficulties in obtaining the optical surface. In this study, the possibility for obtaining optically graded surfaces of titanium alloys by ultra-precision polishing was investigated. Before the ultra-precision polishing, ultra-precision turning with a single point diamond tool was used to get all sample surfaces. But, titanium alloy is difficult to obtain good surface quality by ultra-precision diamond turning. The samples results confirmed that most of the surface roughness values are higher than 30 nm. In order to explore the polishing process, a large number of ultra-precision polishing experiments were conducted. In addition, the effects of different ultra-precision polishing parameters on the surface profiles of titanium alloy Ti6Al4V were investigated in depth. The results show that the average values of surface roughness of titanium alloy parts with ultra-precision turning can be further reduced by 70% or so by ultra-precision polishing. Using a reasonable combination of high spindle speed and large cutting depth, the value of surface roughness can even be lower than 2 nm.

## 1. Introduction

Titanium alloys are mainly applied in the sports, ocean and aviation fields owing to the significant properties thereof, including high specific-strength, corrosion resistance, and optimal biocompatibility [1–4]. In recent years, titanium alloys have been considered to have wide application prospects in the fields of ultra-precision, such as optical and biological fields [5–8]. Pornsin-sirirak [5] presented the first MEMS-based wing technology using Ti6Al-4V alloy as a wingframe with a wing weight of 0.3g. Wieding [6] evaluated the capability of the Ti6Al4V alloy as the bone scaffold material by mechanical and biomechanical testing. From the perspective of biological applications, Liu [7] discussed various surface modification techniques of titanium alloys. Wójcik [8] used the multi-sensor to measure the surface texture in the precision part of the titanium alloy.

An increasing amount of attention has been paid to the ultra-precision cutting of various titanium alloys [9–17]. When using the diamond tool in ultra-precision turning, Colafemina [9] analyzed the surface quality of Ti6Al4V alloy and Ti. Zareena [10] investigated the tool wear mechanisms and presented the method for extending tool life during the ultra-precision cutting of Ti6Al4V alloy. Sakamoto [11] analyzed the effect of cutting fluid on surface roughness formed by ultra-precision cutting of titanium metal with the coated carbide tool. Shinozaki [12] used the method of ultra-precision machining with diamond tool in an attempt to cut titanium alloys. Bai [13] investigated the influence of the ultra-precision cutting on the mechanical properties and microstructure of titanium alloys. Yip [14] surveyed the brittle and ductile transition phenomenon of titanium alloys during the ultra-precision turning process with a single point diamond tool. Xiong [15] combined simulations and experiments to analyze the cutting mechanism of titanium alloys during ultra-precision cutting. Hu [16] explored the wear mechanism of a diamond tool in the ultra-precision machining of Ti6Al4V alloy. Kwak [17] used a nanopositioning method to research the ultra-precision machining of Ti6Al4V alloy.

Despite such findings, titanium alloys have also been found to have poor cutting performance in the ultra-precision machining process. There are considerable difficulties in obtaining the optical surface for titanium alloy materials using the ultra-precision cutting method, and thus, numerous methods have been presented for improving the cutting performance of titanium alloys during the ultra-precision cutting process [18–21]. For reducing the cutting difficulty of Ti6Al4V alloy during the ultra-precision cutting process, Lou [18] adopted the electro-pulsing treatment to change the surface mechanical properties. Yip [19] added one magnetic field assisted system into the ultra-precision cutting to guarantee the sustainable cutting of Ti6Al4V alloy. To obtain a better cutting quality, Kazuya [20] used the ultra-high-speed spindle in the ultra-precision cutting of the titanium alloy. Hu [21] used the ultrasonic vibration method to improve the cutting quality of titanium alloy in the ultra-precision turning.

At present, the method of ultra-precision polishing is considered to be the primary method for obtaining a high-quality surface with nanoscale surface roughness. The ultra-precision polishing technique can remove burrs and cracks and reduce the form error and surface roughness [22–25]. Li [22] explored the evolution mechanism of molten pool formed during polishing process and studied the effect of polishing on the cutting of Ti6Al4V alloy with a continuous wave (CW) fiber laser under the top-hat distributed heat source. Song [23] used the ultraviolet-induced (UV-induced) nanoparticle colloid jet machining to carry out the ultra-precision polishing experiment for improving the surface quality of Ti-6Al-4V alloy. Liang [24] adopted the method of chemical mechanical polishing (CMP) to provide a new way for the nano-scale surface of Ti alloys. Jwa [25] proposed the chemistry enhanced shear thickening polishing (C-STP) to obtain the great surface quality of Ti-6Al-4V alloy and achieve high efficiency in the polishing process.

Aiming at the problem that it is difficult to obtain good cutting quality in ultra-precision cutting of titanium alloy, ultra-precision polishing is adopted to further improve the surface quality. Up to now, there has been a scarcity of research on the ultraprecision polishing of titanium alloys. To explore the possibility of obtaining the optical surface, the ultra-precision polishing process of titanium alloy needs more in-depth analysis and research. A series of experiments of ultra-precision polishing were conducted using the NC ultra-precision polishing machine. The research confirmed that the ultra-precision polishing method can improve greatly surface quality of Ti6Al4V alloy using the reasonable parameters. In addition, the polishing parameters have the different effect on the surface roughness of titanium alloy Ti6Al4V. The results show that higher spindle speed and larger polishing depth are more beneficial to reduce the surface roughness.

## 2. Setup of experiment

**Fig 1:**
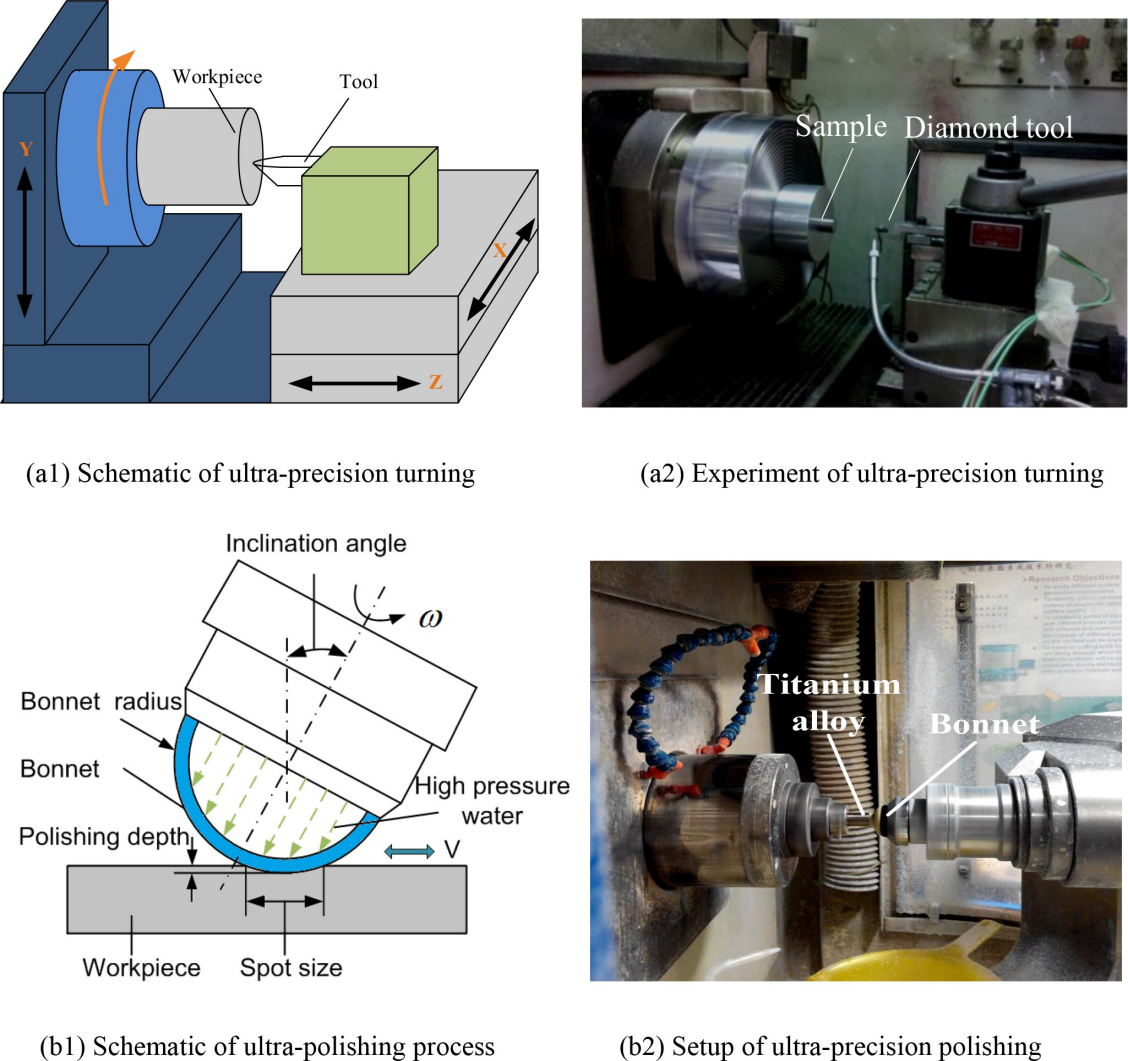
Ultra-precision turning and polishing of titanium alloy.

In the present study, the material type was the typical titanium alloy Ti6Al4V. Before the ultra-precision polishing experiments, all samples of Ti6Al4V alloy needed to be cut by ultra-precision turning with a single-point diamond tool. The ultra-precision turning experiments were conducted on the numerical control ultra-precision machine Nanoform 300. The Ti6Al4V samples were rod-shaped. The dimensions of the samples were: 14 mm diameter and 40 mm length. The schematic and experimental setup of the ultra-precision turning for titanium alloy is shown in Fig 1A. The single-point diamond tool was adopted during the turning experiments.

To explore the effect of polishing parameters on the surface roughness of titanium alloy, a number of ultra-precision polishing experiments for Ti6Al4V alloy were conducted on the ultra-precision polishing machine (Zeeko IRP200) after the samples of Ti6Al4V alloy were cut by ultra-precision turning. Fig 1B shows the ultra-precision polishing schematic and practical experiment of Ti6Al4V alloy. The material of the bonnet was rubber. The polishing solution used in the ultra-precision polishing experiments for titanium alloy was SiO_2_ slurry and the average diameter of the SiO_2_ particles was about 75 nm.

To obtain the surface roughness of samples after the ultra-precision turning and polishing, an optical measurement machine (WYKONT8000) was used. The measure position of surface roughness obtained by the ultra-precision and turning polishing was located at 6 mm of the sample center. The detailed turning and polishing parameters of ultra-precision turning tests are listed in Table 1. The cutting parameters in this paper are selected based on the parameters in the references [15,16,21].

**Table 1:** Ultra-precision turning and polishing parameters.

	Parameters	Values
Turning	Depth of cut Feeding speed	4 μm 9 mm/min
	Spindle speed	1200 rpm
Polishing	Cooling Diamond tool Bonnet radius (mm) Spindle speed (rpm) Polishing depth (um) Feeding speed (mm/min) Inclination angle (^o^) Gas Pressure (bar) Polishing time (min)	Oil mist rake angle 0^o^, clearance angle 15^o^ 10 400,800,1200, 1600 0.5,1,1.5,2 3,6,9,12 5,10,15,20 0.5,1,1.5,2 2,4,6,8

**Fig 2:**
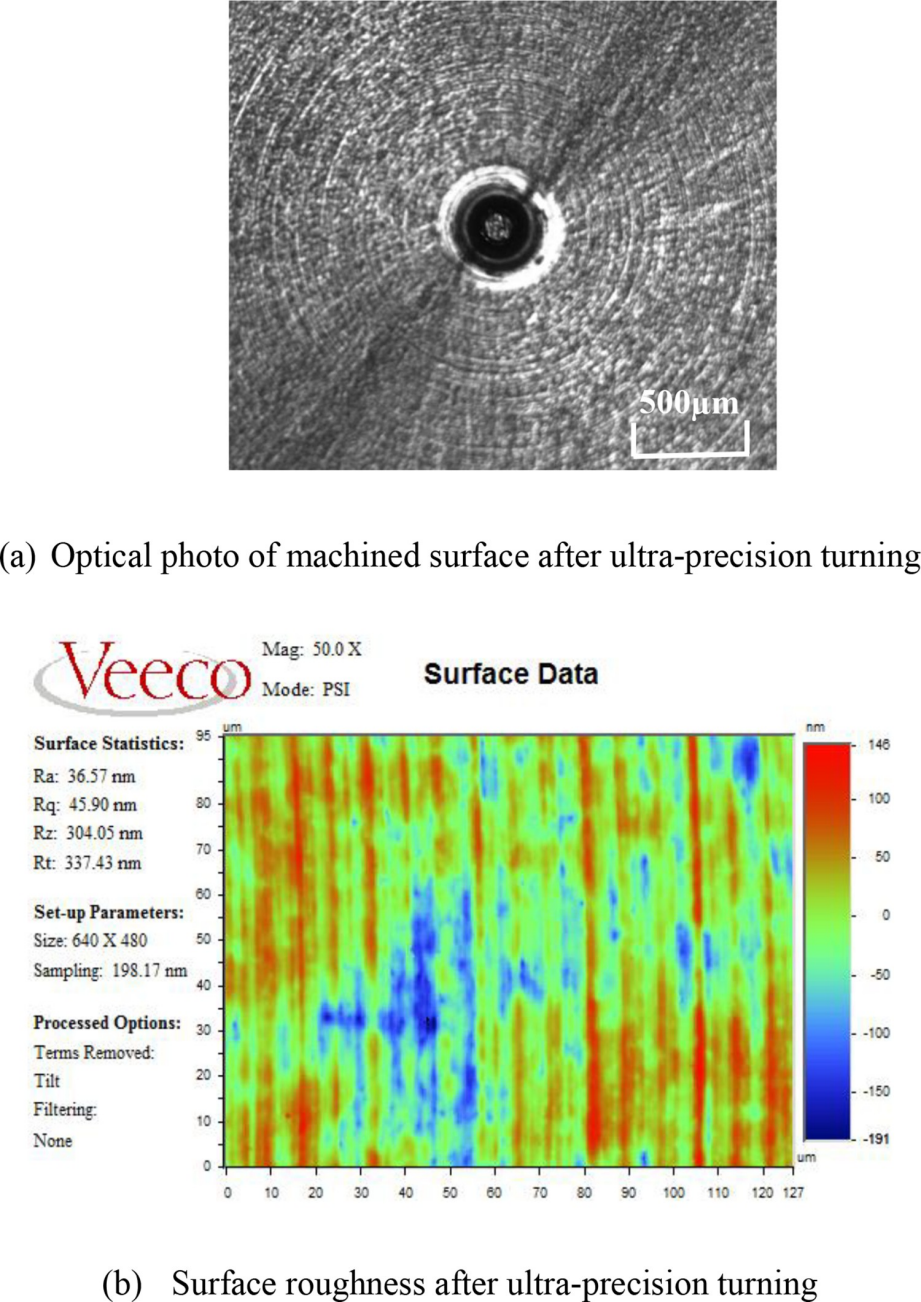
Surface profile of one of Ti6Al4V samples.

Under the same cutting parameters, a batch of titanium alloy samples were machined by the ultra-precision cutting. All roughness values of Ti6Al4V samples after the ultra-precision turning were between 30 and 40 nm. The results show that the surface roughness of Ti6Al4V samples could hardly meet the requirements of the optical surface by ultra-precision turning. Additionally, the samples required further processing. Fig 2 presents the optical photo and 2D surface profile of the machined surface of one Ti6Al4V sample after ultra-precision turning. The tool marks are clearly visible in the figure, and the surface roughness was about 36 nm and the value is also close to that in the literature [15,16]. This figure shows that the optical surface quality is difficult to be achieved by the ultra-precision turning. This is mainly due to the strong viscosity of titanium alloy, high chemical activity, resulting in poor machinability. In the cutting process of titanium alloy, the diamond tool wear fast and the cutting quality is difficult to be guaranteed.

As a finishing process, polishing is usually performed to reduce the surface roughness and obtain a smooth surface. Preston [26] presented a prediction model of material removal for the polishing process and the model is:

Δh(x,y)=k⋅v(x,y)⋅p(x,y) (1)

where Δ*h*(*x, y*) is the material removal in unit time, *k* is the coefficient of Preston which is relevant to the workpiece, bonnet, slurry and the environment temperature, *v(x*,*y)* is the surface velocity between workpiece and bonnet; and *p(x*,*y)* is the polishing pressure on the workpiece.

**Fig 3:**
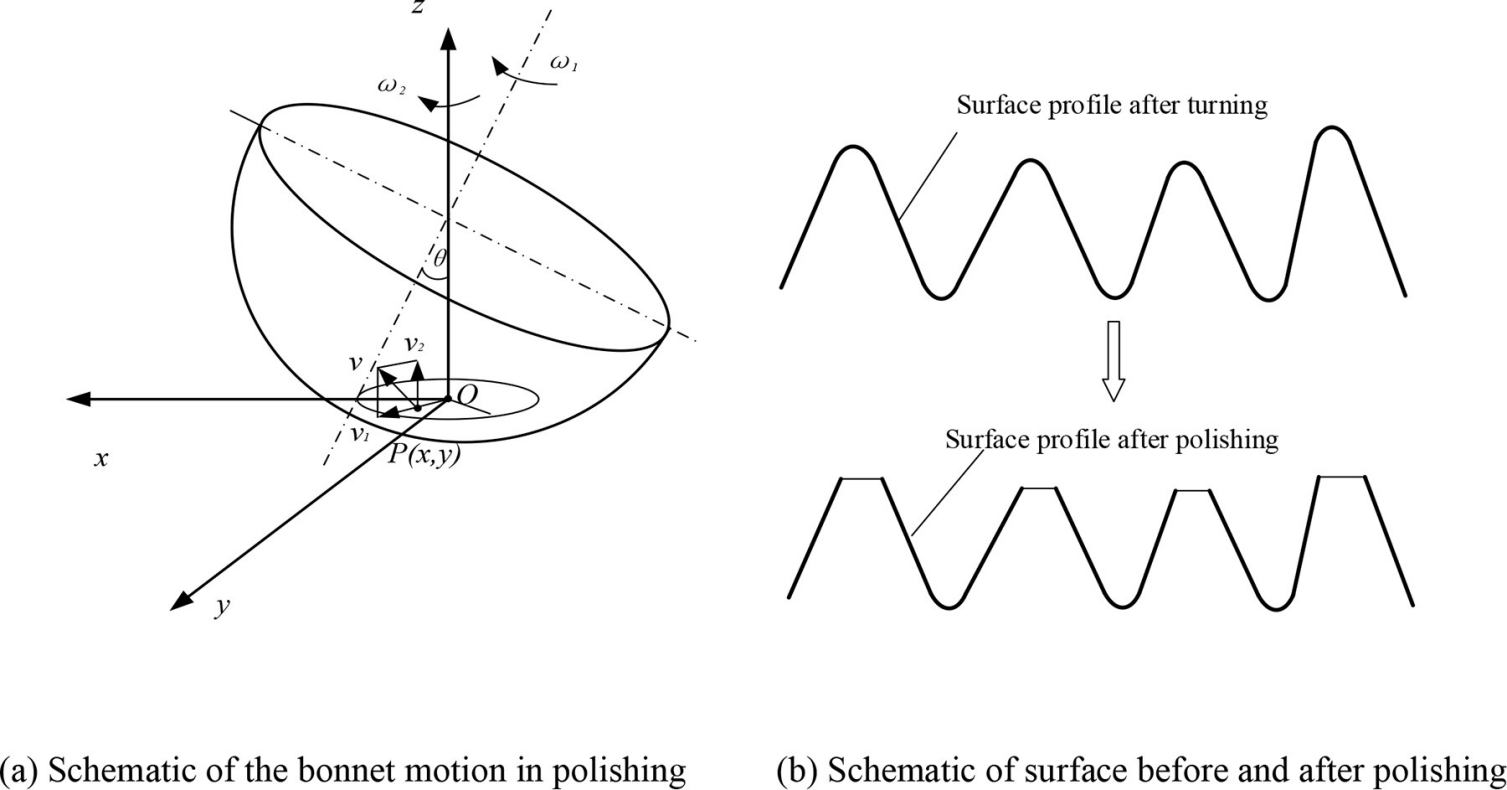
Schematic of polishing process.

If the average value of material removal in unit time *T* is defined to be *R(x*,*y)*, and the bonnet influence function and the revolution period of the polishing bonnet is defined to be *T = 2π/ω*, the function of material removal *R(r)* can be defined to be:

$$R(r)=\frac{1}{T}\int_{0}^{T}k\cdot v(x,y)\cdot p(x,y)dt=\frac{k}{2\pi }\int_{-\theta }^{\theta }v(x,y)\cdot p(x,y)d\theta$$ (2)

where *ω* is the angular velocity of the bonnet; and *θ* is the rotation angle of the bonnet. The function of material removal *R(r)* can be determined through Eq 2 when the distribution of surface velocity *v(x*,*y)* and the distribution of pressure *p(x*,*y)* are achieved. Fig 3A shows the schematic of the bonnet motion in the polishing process [27]. The burrs, cracks, form error and other surface flaw that occur in the ultra-precision turning process could be reduced by the ultra-precision polishing. Fig 3B shows the schematic of the material removal in polishing.

## 3. Results and analysis

**Fig 4:**
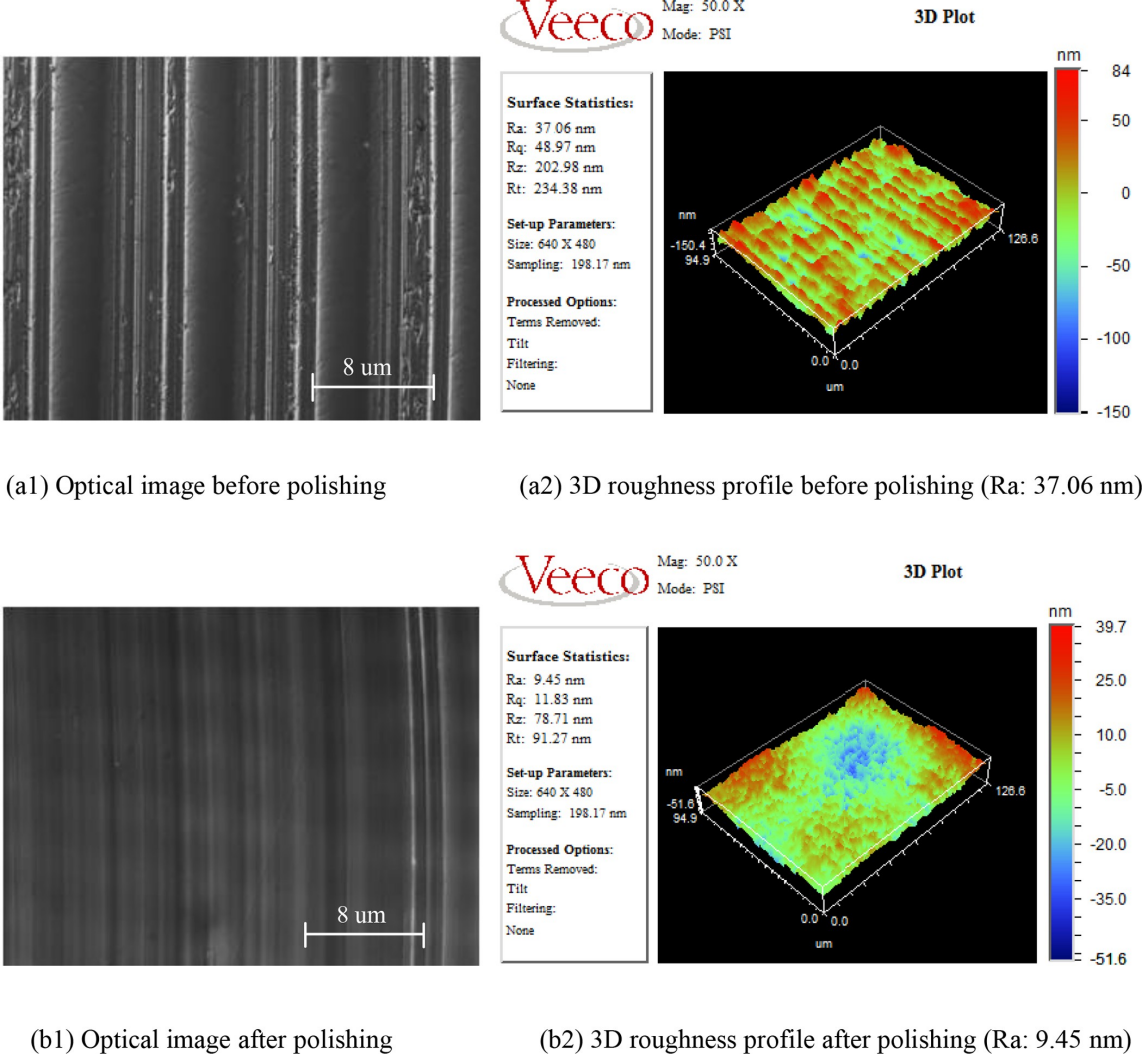
Comparison of surface profiles of one Ti6Al4V sample before and after polishing.

All titanium alloy samples were measured after the ultra-precision turning and polishing by the WYKONT8000. The optical surface morphology photo and 3D roughness profile of one titanium alloy sample are shown in Fig 4. The parameters of ultra-precision polishing were as follows: inclination angle of 10o, spindle speed of 1200 r/min, depth of polish of 1 μm, feeding speed of 9 mm/min, and pressure of 1 bar. From Fig 4, an observation can be made that the tool marks on the machined surface caused by the single-point diamond tool became shallow and the surface became smoother through the ultra-precision polishing. Further, the values of the surface roughness changed from above 36 nm to below 10 nm. The results show that it is difficult to obtain good surface quality by ultra-precision turning, and only ultra-precision polishing of titanium alloy can obtain optical surface. At the same time, the results of this study are consistent with those in the literatures [28–31]. Yasui [28] successfully reduced the surface roughness value of titanium alloy from 200 nm to 84 nm by using low ultra-precision turning speed. Additionally, Yasui [29] got a 60 nm surface roughness with TiCN coated carbide tool in the ultra-precision turning of titanium alloy. Kwang-Pyo [30] obtained 2.693 nm surface roughness of titanium alloy using the ultra-precision polishing method. Zhang [31] used the different polishing path to get the better surface quality in the ultra-precision polishing of titanium alloy and the surface roughness is less than 10 nm.

**Fig 5:**
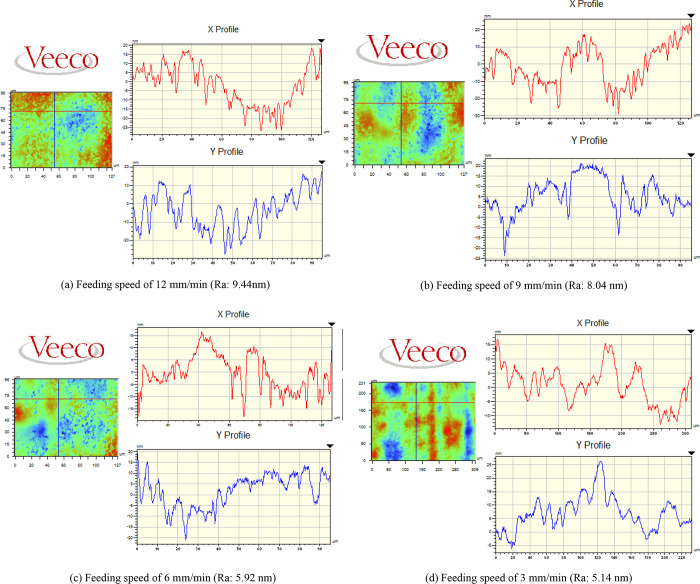
Roughness under different feeding speeds.

Fig 5 shows the 2D surface roughness of Ti6Al4V alloy after polishing under different feeding speeds. In the polishing experiments, the other polishing parameters were as follows: spindle speed of 1200 r/min, inclination angle of 10^o^, depth of polish of 1 μm, and pressure of 1 bar. From Fig 5, all values of surface roughness (Ra) of the Ti6Al4V specimens decreased significantly and fell below 10 nm after ultra-precision polishing. The results reveal that the roughness values of the Ti6Al4V samples reached the optical demands. However, the values of surface roughness had no obvious differences under different feeding speeds. In addition, the roughness values of the four Ti6Al4V samples were greater than 5 nm. As can be seen from the X and Y directions of the surface profile, there are no excessive peaks in the profile and most of the residual height has been removed by the ultra-precision polishing process. At the same time, the results show that ultra-precision polishing can reduce the surface roughness, but the feed speed is not the main parameter affecting the surface quality. When the cutting depth is larger, the contact area between the polishing bonnet and workpiece is larger, and the effect of feed speed is not obvious. The results are in good agreement with those in the literature [31].

**Fig 6:**
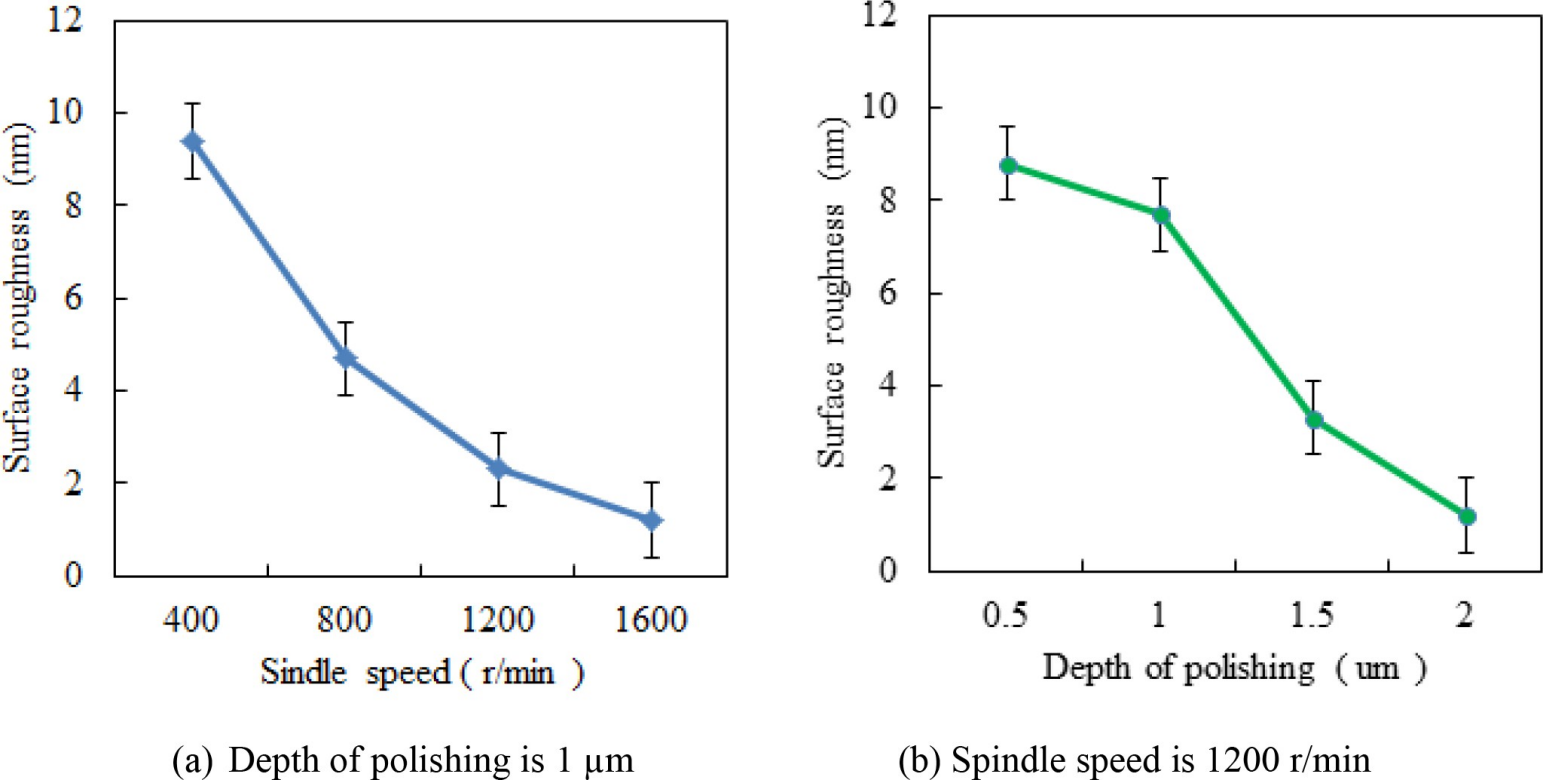
Influence of polishing depth and spindle speed on surface roughness.

Fig 6 shows the change of surface roughness obtained in the polishing tests with different depths of polishing and spindle speeds. The other polishing parameters were as follows: feeding speed of 3 mm/min, inclination angle of 10^o^, and pressure of 1 bar. Fig 6 shows that the surface roughness value decreased as the depth of polishing and spindle speed increased. The tests results confirm that a higher spindle speed and a larger depth of cutting can facilitate a smoother surface for Ti6Al4V alloy during the ultra-precision polishing process. The results show that the spindle speed and cutting depth are the main factors affecting the surface quality, and the combination of high spindle speed and cutting depth can greatly reduce the surface roughness, and the surface roughness can even be less than 2 nm. This is mainly because the increasing of the cutting depth will increase the contacted surface between bonnet and workpiece, while the increase of spindle speed will increase the velocity of the abrasive particles, then the friction action of the polishing bonnet and the abrasive particles with the cut surface will be strengthened, and the effect of material removal is enhanced and the polishing quality is improved.

**Fig 7:**
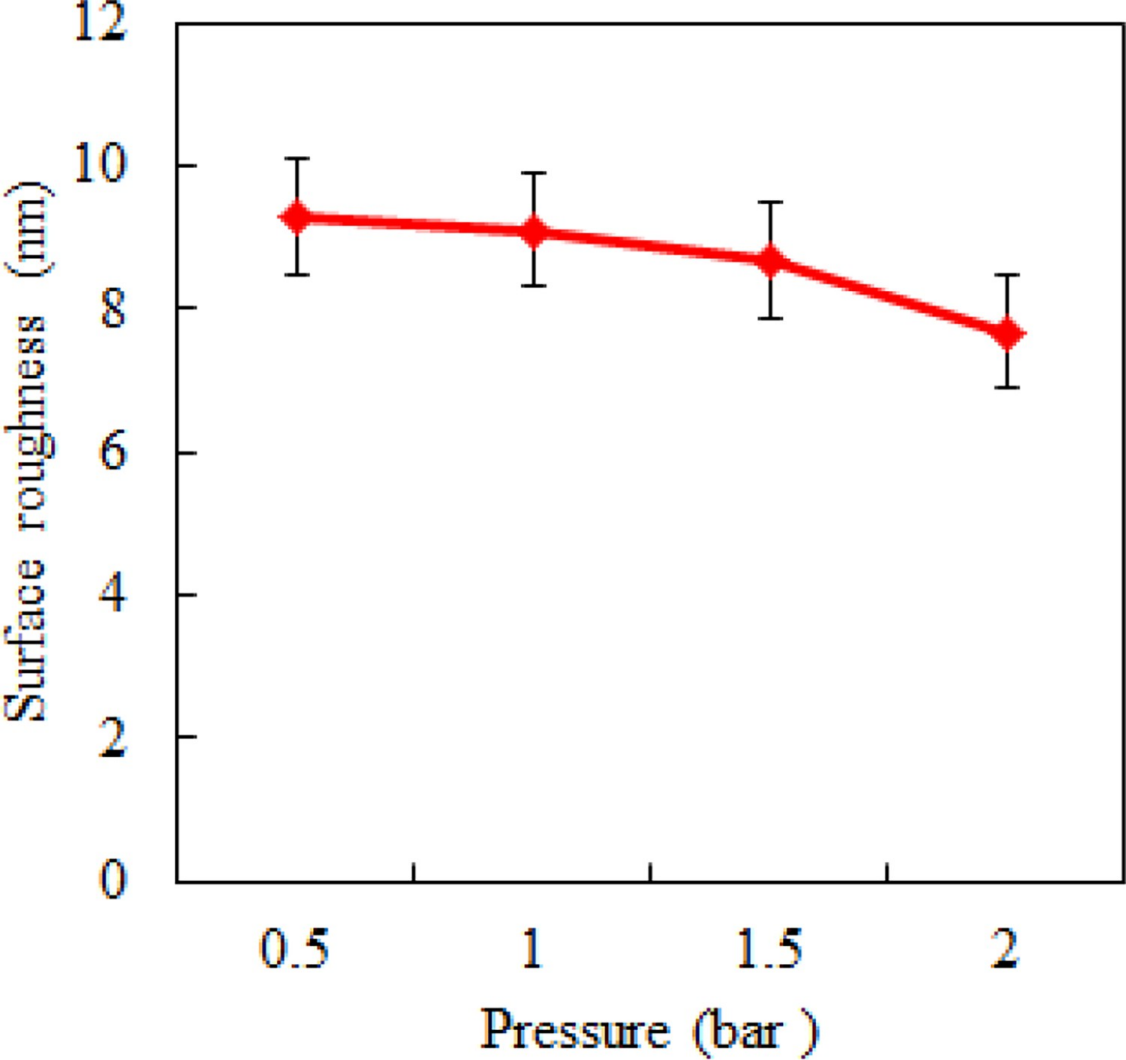
Effect of pressure on surface roughness.

The variation law of surface roughness of Ti6Al4V alloy with pressure is shown in Fig 7. The other polishing parameters were as follows: spindle speed of 1200 r/min, inclination angle of 10^o^, polishing depth of 1 μm, and feeding speed of 6 mm/min. As can be seen from Fig 7, the pressure in the ultra-precision polishing had a small influence on the values of surface roughness of titanium alloy Ti6Al4V. The results show that high pressure can reduce the surface roughness value, but the effect of pressure is not the main factor affecting the surface quality in the ultra-precision polishing of titanium alloy.

**Fig 8:**
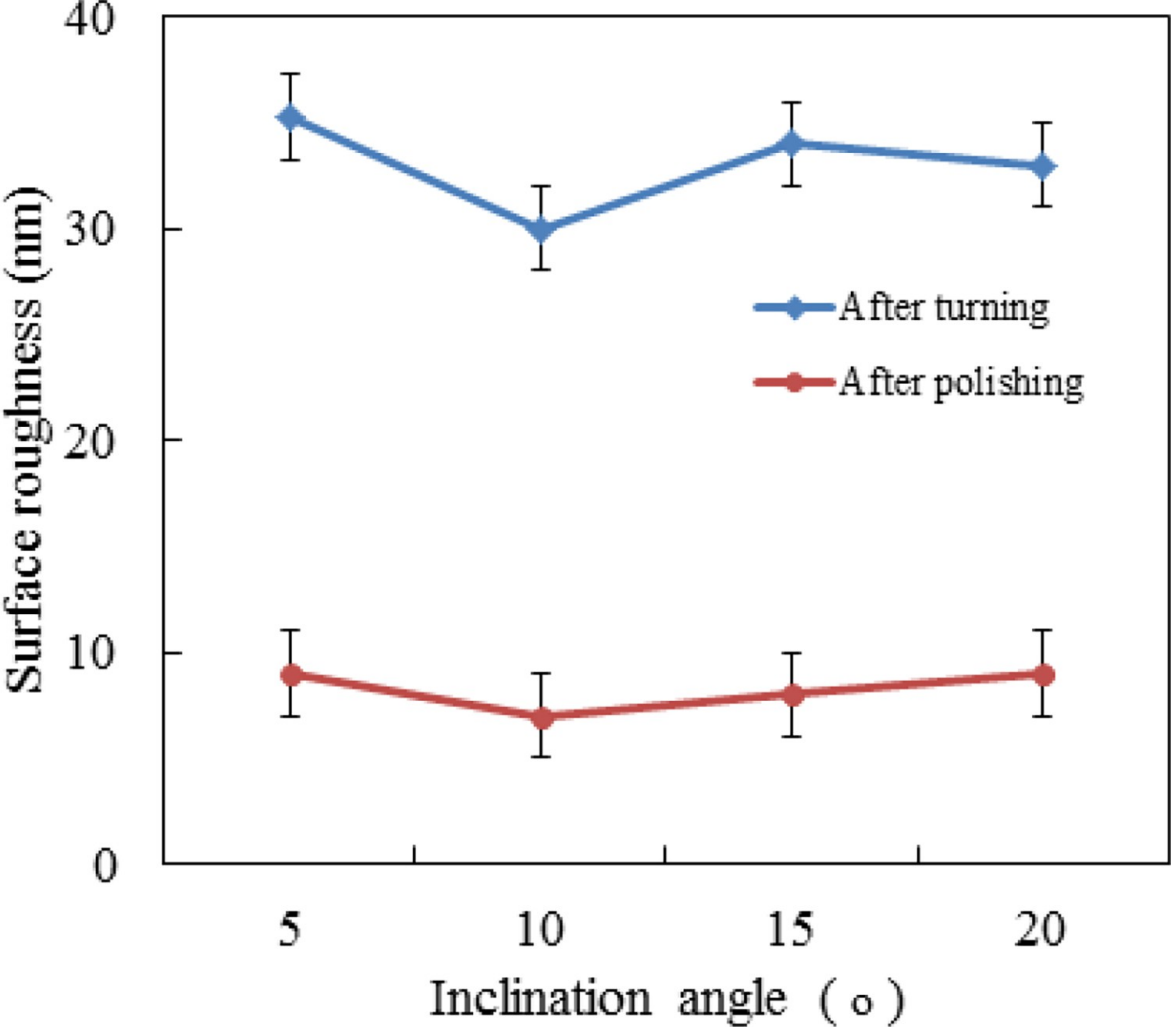
Effect of inclination angle on roughness.

The influence of inclination angle on the values of roughness of titanium alloy Ti6Al4V is shown in Fig 8. The other polishing parameters were as follows: spindle speed of 1200 r/min, pressure of 1 bar, polishing depth of 1 μm, and feeding speed of 6 mm/min. It can be seen from Fig 8, the roughness slightly changed as the inclination angle increased. The result shows that polishing can greatly reduce the surface roughness value, but the increase of inclination angle cannot improve the polishing quality significantly.

**Fig 9:**
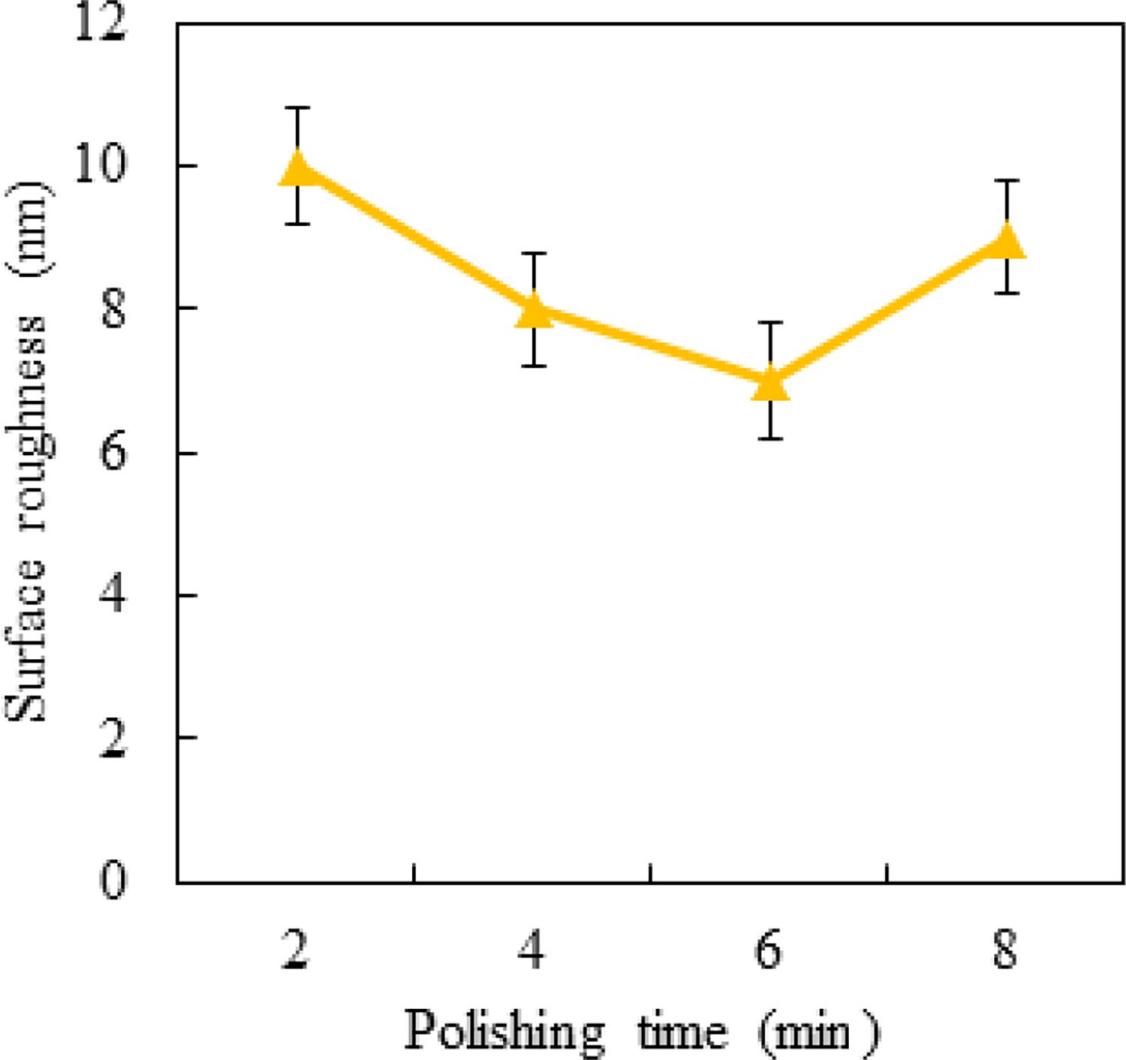
Effect of polishing time on roughness.

Fig 9 shows the effect of polishing time on the surface roughness of titanium alloy Ti6Al4V. The other polishing parameters were as follows: spindle speed of 1200 r/min, pressure of 1 bar, polishing depth of 1 μm, inclination angle of 10o, and feeding speed of 6 mm/min. From Fig 9, an observation can be made that a longer polishing time did not result in a better surface quality. The results show that reasonable polishing time can improve the surface quality, but too long polishing time can increase the value of surface roughness. The results show that too long polishing time will lead to over-polishing.

## 4. Conclusions

An increasing amount of attention has been given to the ultra-precision cutting of titanium alloys. However, the bad machinability of titanium alloys added the difficulty of ultra-precision machining. There are difficulties in obtaining the optical surface by using ultra-precision diamond cutting. In the present study, the ultra-precision processing used to improve the surface quality of titanium alloys. A number of ultra-precision polishing experiments of Ti6Al4V alloy were conducted after ultra-precision turning for exploring the ultra-precision polishing mechanism of Ti6Al4V alloy. The influences of various polishing parameters on the surface profiles of Ti6Al4V alloy were analyzed in-depth. At the same time, the results are compared with those in other literatures and the results are consistent. The results of the study are as follows:

1. It is difficult to guarantee the surface quality of titanium alloy by ultra-precision turning, most of the surface roughness values of samples are above the 30 nm.
2. Ultra-precision polishing can greatly improve the surface quality of titanium alloy, and the surface roughness value can be below 2 nm when using reasonable polishing parameters.
3. The experimental results demonstrate that the polishing depth and the spindle speed had significant effects on the roughness value, while feeding-speed, pressure and inclination angle had little effect on the surface roughness. In addition, the polishing time is neither too long nor too short.
